# Google Earth Engine as Multi-Sensor Open-Source Tool for Supporting the Preservation of Archaeological Areas: The Case Study of Flood and Fire Mapping in Metaponto, Italy

**DOI:** 10.3390/s21051791

**Published:** 2021-03-04

**Authors:** Carmen Fattore, Nicodemo Abate, Farid Faridani, Nicola Masini, Rosa Lasaponara

**Affiliations:** 1 Institute of Methodologies for Environmental Analysis, Italian National Research Council, C.da Santa Loja, Tito Scalo, 85050 Potenza, Italy; carmen.fattore@imaa.cnr.it (C.F.); farid.faridani@unibas.it (F.F.); 2 DICEM (Dipartimento delle Culture Europee e del Mediterraneo), University of Basilicata, Via Nazario Sauro, 85100 Potenza, Italy; nicola.masini@cnr.it; 3 Institute of Heritage Science, Italian National Research Council, C.da Santa Loja, Tito Scalo, 85050 Potenza, Italy

**Keywords:** Google Earth Engine, environment risks, forest fire, flood, Sentinel-1, Sentinel-2, Landsat 8, MODIS, cultural heritage

## Abstract

In recent years, the impact of Climate change, anthropogenic and natural hazards (such as earthquakes, landslides, floods, tsunamis, fires) has dramatically increased and adversely affected modern and past human buildings including outstanding cultural properties and UNESCO heritage sites. Research about protection/monitoring of cultural heritage is crucial to preserve our cultural properties and (with them also) our history and identity. This paper is focused on the use of the open-source Google Earth Engine tool herein used to analyze flood and fire events which affected the area of Metaponto (southern Italy), near the homonymous Greek-Roman archaeological site. The use of the Google Earth Engine has allowed the supervised and unsupervised classification of areas affected by flooding (2013–2020) and fire (2017) in the past years, obtaining remarkable results and useful information for setting up strategies to mitigate damage and support the preservation of areas and landscape rich in cultural and natural heritage.

## 1. Introduction

Since the beginning of the new millennium, public awareness of world cultural heritage (CH) has increased. This is certainly due to several phenomena that have involved the whole society, such as the diffusion of information through direct channels (e.g., social media), or the possibility for everyone to travel around the world. CH has been recognized as an active source of social and economic development, as well as identity. An example is the importance given to CH within the UNESCO’s SDGs (United Nations Educational, Scientific and Cultural Organization-Sustainable Development Goals) in the framework of the Agenda 2030. At the same time, the world’s increased attention to its CH has been matched by increased attention to the loss of CH itself. In recent years, the CH’s causes of destruction and risk in different areas of the world have changed significantly compared to the past. Natural risks have increased due to climate change, on a global scale, and anthropogenic risks and degradation phenomena have also increased exponentially, such as those due to: (i) pollution, (ii) heavy agriculture and land-use, (iii) urban sprawl, (iv) war or looting events, and (v) overtourism [1]. In this context, the use of remote sensing (RS) and Earth Observation (EO) technologies can be very supportive in the light of the advancement of satellite data acquisition and processing systems. EO techniques and aerial photography have long been widely used in archaeological research, primarily for the identification of buried or hidden remains [2,3,4,5,6,7]. However, this type of study has focused on the research itself, with the goal of discovery, while in recent years, the trend to apply several EO to CH with different purposes has increased, directly related to the concepts of risk-damage-loss, aimed to preserve and transmit to future generations the world CH. Several techniques have been used and are well described in the literature:

1. the use of historical maps and aerial photos, associated with GIS (Geographic Information System) to understand landscape changes (urban sprawl, land use, subsidence phenomena, and geomorphological variations etc.) over time, for CH risk assessment or management [8,9,10,11,12,13];
2. the use of multisensor and multitemporal satellite data such as declassified optical data [14] and medium-high resolution multispectral data [15,16,17,18,19,20], or SAR (synthetic aperture radar) data [21,22,23,24], for anthropogenic (land use change, destruction, looting) or natural (floods, subsidence, fires, etc.) risk assessment.

Several satellite platforms are currently in orbit and capture a wide variety of data, thus enabling multitemporal, multisensor, and multiscale analyses. Moreover, in recent years, a significant increase in software and hardware technologies has been achieved for processing satellite data, and this is particularly relevant in the era of Big-EO Data. Google Earth Engine (GEE) offers powerful tools and EO data particularly relevant for applications aimed at the prevention, risk monitoring, and damage estimation [25]. For these reasons, GEE was herein chosen as a tool for processing multi-sensor/platform, multi-temporal data used to analyze flood and fire events which affected the area of Metaponto (southern Italy), near the homonymous Greek-Roman archaeological site.

GEE is an open-source tool made available by Google through registration. It is a portal that allows to consult and work simultaneously with dozens of different datasets for a collection of over forty years of data on a global scale. GEE is a powerful high-performance computing tool, it is accessed and controlled through a web-based accessible application programming interface (API) and there is an associated web-based interactive development environment (IDE) that allows quick prototyping and visualization of results [26]. Available datasets include satellite data acquired by several missions, such as: (i) MODIS (Moderate Resolution Imaging Spectrometer); (ii) ALOS (Advanced Land Observing Satellite); (iii) Landsat series; (iv) Sentinels etc. These are complemented by other useful/ancillary data, such as: (i) DTMs (digital terrain models); (ii) shapefiles; (iii) meteorological data; (iv) land cover, etc. [26,27]. GEE, in addition to making operations with several types of sensors extremely easy, allows the same way and time to work with petabytes of data and has changed the concept of work and analysis in the field of remote sensing, and to the big-data approach to the issues. GEE in recent years (2011 to date) has been widely and successfully used in many of the disciplines involving EO techniques due to its potential. According to statistics reported in other studies on GEE, the number of articles on the topic has increased from less than ten articles per year in 2013 to more than 200 in 2019 [28,29].

The number of papers about GEE [26,30,31,32] in recent years has increased exponentially in several fields, such as: (i) forest and vegetation [33,34]; (ii) land use and land cover [35,36,37]; (iii) hydrology [38,39,40]; (iv) ecosystems and sustainability [41]; (v) agriculture [42,43,44,45]; (vi) climate [46]; (vii) urban sprawl [47]; (viii) hazards [48,49]; (ix) cultural heritage [50]. The use of GEE has allowed multiple tools to be created and shared for free. Some examples can be found on the GEE website [51].

Google Earth Engine has been herein used to monitor the area of Metaponto (see Section 2.1.1.), important for the presence of archaeological remains of different eras, and for the fire and flood events that have affected it in recent years (see Section 2.1.2.). Data from 2013 to 2020 acquired by several satellite platforms, Sentinel 1 [52], Sentinel 2 [53], Landsat 8 [54], and MODIS [55] were used in a single tool (GEE) for this analysis. All the data were processed using JavaScript via the GEE web portal and each process took a few tens of seconds, in full cloud-computation style.

## 2. Materials and Methods

### 2.1. Study Area

**Figure 1:**
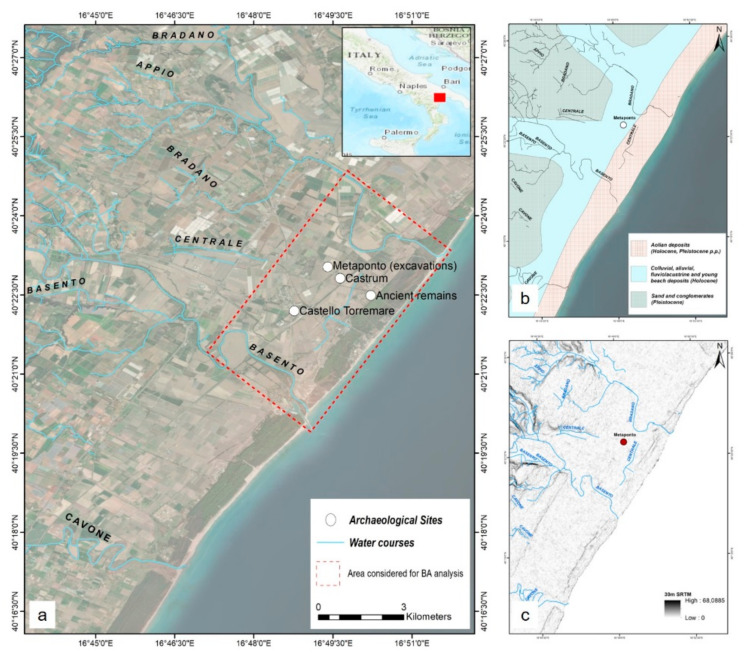
(**a**) Study area; (**b**) Geological map; (**c**) 30 m SRTM (Shuttle Radar Topography Mission).

The study area is located in southern Italy, in Basilicata, in the Gulf of Taranto, on the Ionian Sea. It is enclosed between the Cavone (south-west) and Bradano (north-east) rivers, up to the Appulo-Lucano Apennines in the north-west and the Ionian Sea in the south-west. The choice was made for the importance of the archaeological remains in the territory of the city of Metaponto, in recent years several times at risk due to natural and anthropogenic events (fires, floods, change of the shoreline). The area covers about 132 km^2^ and this extension has been considered for the flood analysis, while for burned area (BA) analysis the area has been reduced near the archaeological site of Metaponto and the pine forest in front of it, for a total of 32 km^2^ (Figure 1).

#### 2.1.1. Metaponto and Its Territory

The area between Cavone and Bradano rivers is of great importance for understanding the historical phenomena of the Lucanian territory and southern Italy. Archaeological studies have found evidence of a period ranging from prehistory to the late Middle Ages. As for many regions of Italy, also the area of Metaponto (Μεταπόντιον) has been of interest due to a multitude of archaeological studies since the 19^th^ century, and the scientific literature on the subject is still enriched by new excavations, survey activities, and publications [56,57,58,59,60,61,62,63]. In addition to archaeological studies, the area has been investigated with RS and EO techniques by several authors and research groups for the monitoring of environmental risk assessment (wildfire, flooding etc.), and for the discovery of buried archaeological remains [64,65,66,67,68,69].

From a geological point of view, the Metaponto plain falls within the so-called “Fossa Bradanica”, a basin of sedimentation, filled by a Pliocene-Pleistocene sedimentary succession up to 2–3 km. The geological stratigraphy, consisting of the alternation of long-term events linked to the land-water system (tectonic evolution, glacio-eustatic variations, coastal evolution, changes in the water regime), can be summarized as: (i) subappennine clays; (ii) terraced marine deposits; (iii) alluvial and coastal deposits [64,65,70,71] (Figure 1b).

From a geomorphological point of view, the area looks like a plateau with medium and low altitude relief, consisting of sand dunes, which slopes down to the sea. The area has an extensive network of canals, waterways, and rills [71] (Figure 1c).

The continuous occupation of the area is mainly due to its proximity to water sources and the possibility of using the flat land for cultivation, and the hinterland for pasture, as well as the proximity to the sea. However, the area had disadvantages such as easy flooding due to the aquifer, and malarial fever resulting from the unhealthy environment. It was solved by the ancient peoples by occupying raised terraces, creating infrastructure (canals, tributaries, and drainage systems) and reclaiming the areas for agricultural use. The same problem occurred in recent times (first half 20th century) and was solved by several land reclamations/drainage operations during the fascist period [72].

**Figure 2:**
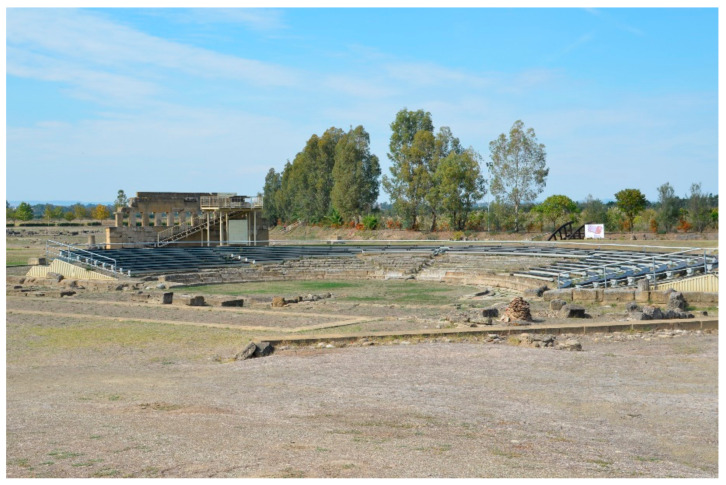
The Theater of Metaponto (Author: AlexanderVanLonn, source: https://commons.wikimedia.org/wiki/File:Metapontum_theater_AvL.JPG (accessed on 4 March 2021), license: CC-BY-SA-3.0).

According to archaeological sources, survey activities, and excavations, the area was occupied starting from the Neolithic period. The archaeological research produced since the 1970s of the 20th century has highlighted the presence of villages and settlements, for the control of the territory and roads, as in the case of San Vito di Pisticci, Termitto, Santa Maria d’Anglona, Incoronata-San Teodoro, Chiaromonte, Noepoli and Timmari, Craco, S. Arcangelo, Ferrandina etc., starting from the Bronze Age [73,74,75,76,77]. To date, the site probably best known and studied is the ancient city of Metaponto. The foundation of the Greek colony is mentioned in several ancient sources, which set the foundation during the late 7th century B.C. [78]. The formation of the Greek-Achean nucleus of Metaponto, is dated to the middle of the 7th. The organization of the city (*polis*, πόλις) and of the territory (*chora*) of the Greek colony had to be articulated, but similar to that of the tradition of the Greek colonies and cities. The city had to occupy an area of about 150 hectares, between the rivers Bradano and Basento, and the sea [79]. The proximity to the sea and the presence of an aquifer close to the surface had to influence the urban planning. The settlers made choices based on this issue since the foundation, with the positioning of the important religious and civil areas (Figure 2) above raised sand dunes, and with the creation of canals and drainage systems (visible from aerial photography and archaeological excavations even today) [80].

**Figure 3:**
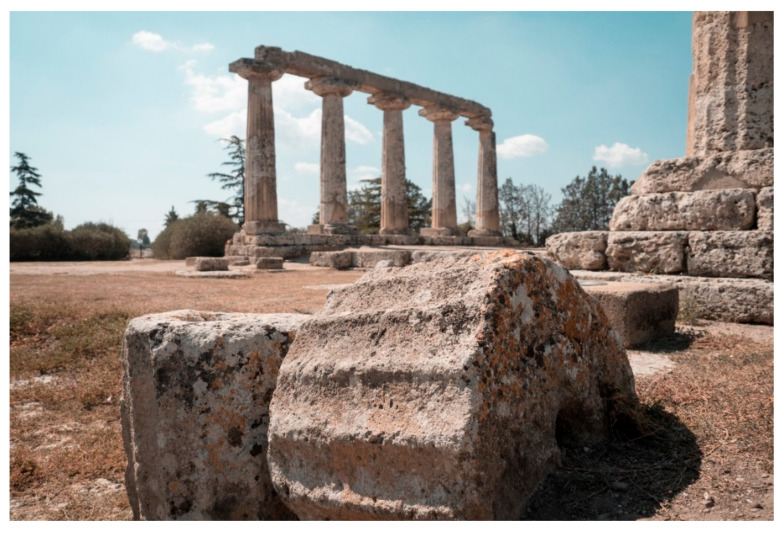
Metaponto: Tavole Palatine (Author: Attilio Bixio, source: https://commons.wikimedia.org/wiki/File:Metaponto_Tavole_Palatine.jpg (accessed on 4 March 2021), license: CC-BY-SA-4.0).

The urban development of the city appears regular, based on the rigid Hippodamian principle of orthogonal main streets, called *platêiai*, and secondary streets, called *stenōpói* [81]. The chora was also developed, as demonstrated by the discovery of numerous farms for agricultural exploitation, and by the presence of important sub-urban sanctuaries, located within the territory of Metapontum, such as the sanctuary of Hera, also known as “Tavole Palatine” (4th century B.C.) [59] (Figure 3).

During the 3rd century B.C., the morphology and management of the Greek colony changed radically. This is due to the events that affected the beginning of the 3rd century B.C. with the expansion of Rome, the rebellion of Taranto, and the subsequent “Wars of Pyrrhus” (280–272 B.C.). After the departure of Pyrrhus, Metaponto was forced to accommodate a Roman garrison within the city, which settled in the area called “*castrum”* (from Latin: military camp) [62]. The *castrum* was built on the eastern side of the city, partially occupying the built-up area and part of the Greek *agorà*, and was connected by a road and channel to the port, located not far away, towards the sea, in Località Mele, in the so-called Lake of Santa Palagina, about one kilometer from the modern shoreline [82,83,84,85,86].

Sources are very poor for the period from the 8th to the 11th century A.D. The most reliable hypothesis is that the early medieval settlement was located near the lake of S. Palagina, now disappeared, between the "Roman" port and the late-medieval settlement of Torre di Mare [87,88]. According to ancient literary sources (e.g., Chronica Monasterii Casinensis, 969 A.D.), the toponym used to indicate the area until the 13th century is that of Civitas Sancte Trinitatis. Only during the 13th century it changed to “Torre di Mare” (Turris Maris) with a new harbor (novus portus), which is still preserved today: “… *civitatis sancte Trinitatis que hodie dicutr Turrimaris*”. Archaeological excavations have shown structures and artifacts dating as far back as the 15th century, however, the area is depicted as a fortified site in frescoes and paintings in the 19th century and is still occupied today [89,90].

#### 2.1.2. State of the Art: Floods and Fires That Have Affected the Area over the Past Decade

The increase in the flood and wildfire events in the Ionian coast of the Basilicata region (southern Italy) has damaged significantly different sectors such as tourism and agriculture.

Lacava et al. [68,91] studied the December 2013 flood event over the Metaponto plain in Basilicata and Puglia regions (southern Italy) by analyzing only-December-month VIIRS and MODIS images using the robust satellite techniques (RST) approach. Their proposed method had the false positive rates of 11.5% over an inundated area extent of about 73 km^2^ and indicated that medium-resolution optical imagery can detect and monitor floods even in small hydrological basins. Manfreda and Samela [92] proposed a DEM-based method based on a new geomorphic index to predict flood extents and inundation depth for quantifying flood damages over large and data-sparse areas with reduced computational effort. The case study for the new procedure was the Bradano River basin located in Basilicata region (southern Italy) and the results demonstrated satisfactory performances (RMSE = 0.335 m) in comparison with the inundation depths obtained by hydraulic simulations.

Bentivenga et al. [65] conducted qualitative/quantitative analyses on hydrometric, pluviometric, and erosion/deposition dynamics of main rivers in the Metaponto plain. Their results show that flooding events in the Metaponto plain are originally caused by extreme precipitations as a result of climate change, though anthropogenic activities such as lacking the cleaning and maintenance of the constructed channel network, have amplified the effects of low-intensity rainfall events and triggering flooding events. Thus, most of the recent floods are localized in large areas far from the river belts due to the failure of the reclamation channels.

**Figure 4:**
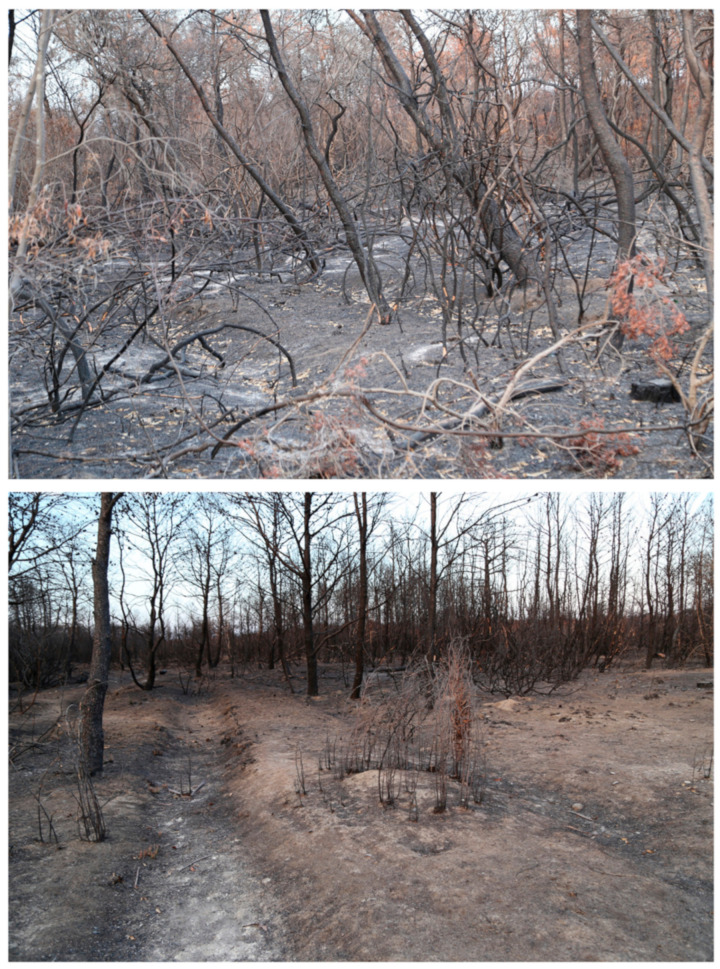
Field survey post-fire event in the pine forest area at Metaponto (Author: Dr. R. Lasponara).

Damage to the economy and risks to the natural and cultural heritage have also been caused by wildfires. The wildfire that affected the pine forest along the coast in 2017 was the subject of study in a recent paper published by Lasaponara and Tucci [93], and it is also the same studied in this paper, with different tools (Figure 4).

Lasaponara and Tucci [93] have used Sentinel-1 A and B SAR data. The images of the pre- and post-fire have been averaged to reduce the visual noise generated by the SAR data and enhance the identification of the burned area anomaly. In further, a statistical analysis based on Getis statistics and an unsupervised classification (ISODATA) have been applied to automatically classify and map the burned area as well as fire severity without the use of fixed thresholds.

### 2.2. Flood Mapping and Monitoring Using Google Earth Engine: State of the Art

In the last decade, the implementation of RS techniques in monitoring flood events and land-surface water bodies has increased by providing real-time, large-scale, and cost-effective information, unlike conventional hydraulic models and in situ measurements [94]. The most common methods to extract water bodies from different RS images are categorized into the following groups: (i) single band density slicing [95], (ii) supervised and unsupervised classification [96,97], and (iii) spectral water indices [98,99,100].

Among these methods, spectral water indices provide more reliable results since they are straightforward, efficient, and require lower computations [101]. McFeeters [102] proposed the normalized difference water index (NDWI) using the green and near infrared (NIR) bands of multispectral optical satellite images to detect open water surfaces, because the water surface absorbs most of the incoming radiation and has low radiation between the visible to infrared wavelengths. Xu [103] demonstrated that NDWI is sensitive to built-up land and over-estimates the water bodies, and replaced the NIR band with the shortwave infrared (SWIR) and introduced the modified normalized difference water index (MNDWI) [94]. Several studies demonstrated the superior efficiency of MNDWI in extracting water surface with greater accuracy than NDWI [98,99,103,104]. The multispectral satellites are classified into two distinguished groups in terms of spatial resolution: (i) the medium-low resolution satellites such as MODIS, Proba-V, or Sentinel-3 with 100–500 m cell size; and (ii) the medium-high resolution satellites such as Sentinel-2 or the Landsat series with 10–30 m cell size [105].

The optical methods suffer from cloud contamination and dense vegetation which impede their application, especially in the tropical regions. Moreover, the NIR reflectance can be higher than the red reflectance over highly turbid water, introducing uncertainty in water detecting indices [106]. On the contrary, the methods based on synthetic aperture radar (SAR) data provide independent data to cloud cover or day and night time, with almost the same spatial resolution as the visible and near-infrared satellite images [94]. The European Space Agency (ESA) has given free access to the Sentinel-1A&B SAR data which has shown strong potential for detecting open water bodies at high spatial resolution [106,107,108,109].

The Google Earth Engine (GEE) cloud computing platform presents a unique opportunity for researchers to have rapid access to analysis-ready data needed for flood monitoring and disaster management. Li et al. [98] developed a flood prevention and response system by implementing the cloud-based GEE platform at each stage of flood events (before, during, and after), the system integrates a range of RS and ancillary datasets including Formosat-2, SAR, and DEM data. This system was successfully tested to manage the Typhoon Soudelor in August 2015 [31]. Vanama et al. [110] proposed a unified framework (GEE4FLOOD) for rapid flood mapping in GEE cloud platform by implementing the Otsu algorithm and processing Sentinel1 SAR data. They tested the GEE4FLOOD on the August 2018 Kerala flood event in India and suggested that GEE can act as an effective tool for mapping flood inundation areas. DeVries et al. [40] presented another algorithm that goes through the archives of Sentinel-1 SAR, Landsat, and other ancillary datasets hosted on the GEE to quickly generate the inundation map of flood events. Their algorithm had acceptable performance in estimating affected areas over three recent flood events in the USA, Greece, and eastern Madagascar, eliminating the need for time-taking data download and pre-processing steps. Singha et al. [111] investigated the spatiotemporal pattern of floods for Bangladesh during 2014–2018 using all the available Sentinel-1 SAR images and the GEE cloud platform and identified the flood-affected paddy rice fields.

### 2.3. Flood Mapping and Monitoring Using Google Earth Engine

To evaluate flood risk, it is necessary to know the stream network of the Metaponto plain where all the main river basins of the Basilicata region (Agri, Basento, Bradano, Cavone, and Sinni) reach to the Ionian Sea. The maximum and minimum hydrometric levels of these rivers normally occur in winter and summer, respectively, with the annual minimum flow rate of 0.5, 0.08, 0.04, 0 (seasonal), and 1.38 m3/sec, respectively. The gauging stations of the Civil Protection Department of Basilicata Region provide this information which are located near the sea [65].

**Figure 5:**
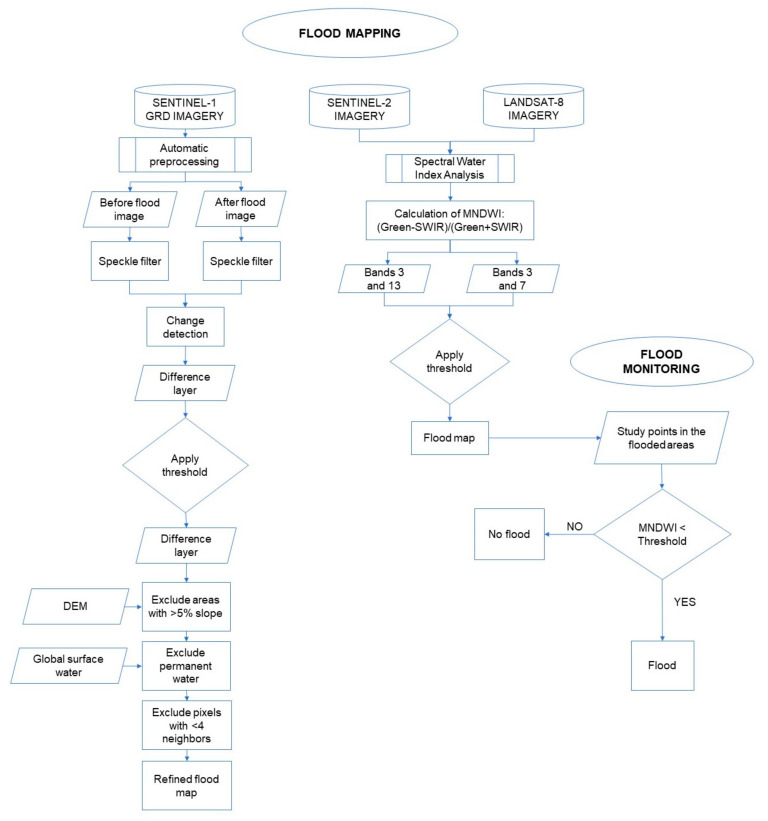
Flowchart for flood mapping and monitoring.

The most common methods to extract water bodies from different RS images are based on the use of water indices: (i) normal difference vegetation index (NDVI), (ii) normal difference water index (NDWI), and (iii) modified normal difference water index (MNDWI), defined in equations 1 to 3:

(1) $$NDVI=\frac{NIR-Red}{NIR+Red}\;,\;$$

(2) $$NDWI=\frac{Green-NIR}{Green+NIR}\;,$$

(3) $$MNDWI=\frac{Green-SWIR}{Green+SWIR},$$

where Green, Red, NIR, and SWIR are the radiance in the green, red, near-infrared, and short-wave infrared bands, corresponding to Landsat 8 bands 3, 4, 5, and 7; and Sentinel 2 bands 3, 4, 8, and 12, respectively. These indices can be calculated by *“image.normalizedDifference([Band1, Band2])”* function in the GEE environment for any multispectral satellite sensor. The generated maps can be classified into flooded and non-flooded areas using an optimum threshold inside the *“Image.lt(threshold)”* GEE function, which produces the most similar results with a reference flood map. This method was implemented for Sentinel2 and Landsat8 imageries in this paper. Another method for detecting flooded areas for Sentinel1 SAR imageries is the classification of difference between two images before and after the events using *“(after_image.divide(before_image)).lt(threshold)”*. The resulting flooded pixels would then be excluded if their slope is bigger than 5%, or if they are not connected to at least 4 more flooded pixels. After identification of flooded areas, the water spectral indices could be monitored for potential flooding behavior in the flood prone areas using *“ui.Chart.image.seriesByRegion({imageCollection.select(’water_index’),regions,reducer:ee.Reducer.mean()});”*. Figure 5 illustrates the workflow of the proposed method.

### 2.4. Forest Fire Mapping and Monitoring Using Google Earth Engine: State of the Art

In recent years, several global-scale BA products have become available [112]. For example, satellite imagery with both low spatial resolution and high temporal resolution, such as the MEdium Resolution Imaging Spectrometer (MERIS), Moderate Resolution Imaging Spectroradiometer (MODIS), and Advanced Very High Resolution Radiometer (AVHRR), are fruitfully exploited near real-time fire detection and monitoring on a global scale [113,114,115]. However, in the case of small fires and fragmented ecosystems, such as in the Mediterranean area, low spatial resolution satellite imagery can significantly undersize the burned area, risking the loss of useful information for post-fire damage assessment. Therefore, medium or high-resolution satellite imagery as Landsat [116,117] or Sentinel-2 (S2) [118] become useful and strategical for BA analysis in a complex and fragmented ecosystem. The systematic acquisition every 5-days over the same area and the free availability of S2 data are fundamental for setting a systematic monitoring system. Several studies have demonstrated the high performance of S2 data for BA analysis [112,119,120,121].

The processing of satellite data to map burned area is usually based on the use of multiple spectral indices to capture the loss caused by fire, and generally resulting in a reduction of chlorophyll absorption and canopy moisture content, causing anomalies in near-infrared (NIR) and mid-infrared (SWIR) reflectance values [121]. One of the most used index for the mapping of burnt area is the normalized burn ratio (NBR) which allows to estimate both the fire extension and burn severity [122]. GEE platform offers specific routines to compute spectral indices and the availability of historical time series of satellite data acquired from several sensors and platforms, totally free. Above all, through appropriate scripting, GEE allows to process huge amount of satellite data in a short time and without the need of supercomputers [30].

Despite these facilities, related both to data availability and processing, up to now, a few studies have been conducted using the GEE platform or similar tools for Monitoring Wildfires for mapping burned areas in (i) the Northeastern Peruvian Amazon using Landsat-8 and Sentinel-2 Imagery [123], (ii) in Italy using S2 [124], and (iii) on the global scale using Landsat Images [125,126,127,128].

### 2.5. Forest Fire Mapping and Monitoring Using Google Earth Engine Tools

**Figure 6:**
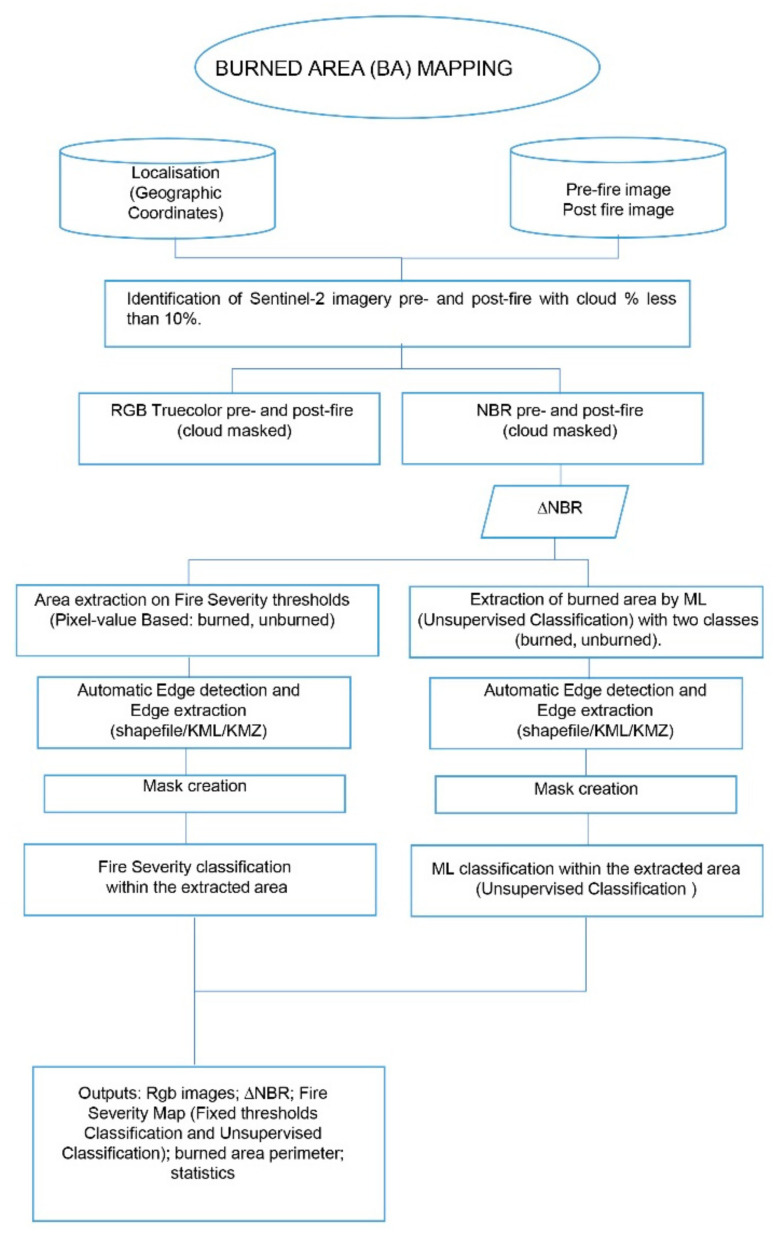
Flowchart for Burned Area (BA) mapping.

Medium spatial resolution satellite data available in the GEE platform were used for this paper, such as bottom of atmosphere (BOA) multispectral image collection as Sentinel-2 level-2A (ID: COPERNICUS/S2_SR) [123,124]. Sentinel-2 satellites acquire 13 spectral bands with spatial resolutions that range from 10 to 60 m. The spectral channels include four bands at 10 m spatial resolution, six bands at 20 m spatial resolution, and three bands at 60 m spatial resolution. S2 products are available as elemental granules, also called tiles (100 km^2^) [129]. The operations are summarized according to the scheme in Figure 6.

The fire event considered for this study is one that occurred on the 13 July 2017, and affected a quite homogeneous, flat area (mainly covered by *Pinus halepensis*) located between the archaeological area and the Ionian coast. To map the area affected by fire using the GEE procedure, the first step was to identify the location (to set the area to be investigated) and date when the fire occurred to search for the cloud free pre- and post-fire image. The identification of cloud free data/ images is a fundamental step to avoid misclassification and incorrectly map the extension of the fire and its severity [130,131]. GEE offers specific script, based on the pixel quality assessment, to mask clouds, cloud shadows and snow, water, etc. Cloud cover can drastically reduce the frequency of observation in the visible/infrared domain and significantly change the spectral reflectance response. Moreover, to avoid the identification of false-positives due to the agricultural cycle, urban green arrangement or other similar events, an additional mask was applied to discriminate the forest/vegetated area from areas dedicated to other human activities, according to the scheme of the Corine Land Cover 2018 (CLC 2018). The cloud free images were then merged by date trying to minimize the extent and impact on the signal of clouds and snow. In addition, the mosaics were clipped in the area of interest and further processed computing the normalized burn ratio (NBR) index calculated as the normalized difference between the NIR and SWIR bands (4) [132]:

(4) $$NBR=\frac{NIR-SWIR}{NIR+SWIR}\;.\;$$

NBR is considered to be very effective in the estimation of the identification of fire affected areas, because it is particularly responsive to changes in the amount of living green vegetation, moisture content, and soil conditions that generally occur after the fire. For our purpose, the difference (∆NBR) between the NBR resulting from the pre- and post-fire images was calculated as (5):

(5) ΔNBR=NBRprefire−NBRpostfire .

∆NBR is considered to be more performant and effective to discriminate fire severity, especially if it is jointly used with statistical indicators, as in [93] to capture the spatial complexity of features and patterns produced by the fire [118]. ∆NBR map highlights the burned area, characterizing the fire severity levels and provides indications of the changes that the fire induced in terms of biomass loss, carbon release, and smoke production.

Table 1 shows the different levels of ∆NBR values suggested by the United States Geological Survey (USGS) to categories fire severity.

The burned area was classified (i) following the USGS severity levels as categorized according to the thresholds listed in Table 1, and also (ii) using automated machine learning (ML) methods, which are available among the GEE functions. Classification is a data mining technique useful to find or group data/pixels that have similar characteristics, features, or value. ML unsupervised classification was chosen because it does not require training data [133]. In the algorithm developed in GEE, the Weka k-means algorithm was applied [134]. K-means is a non-hierarchical clustering method that divides data into a specific number of user-defined clusters [133]. In this case, four classes were selected based on severity levels: (i) high-severity, (ii) moderate-high severity, (iii) moderate-low severity, and (iv) low-severity) following the previous study conducted in the area using Sentinel 1. Results from the unsupervised classification was then compared with the severity classification based on the USGS threshold values.

## 3. Results

### 3.1. Flood Mapping

**Figure 7:**
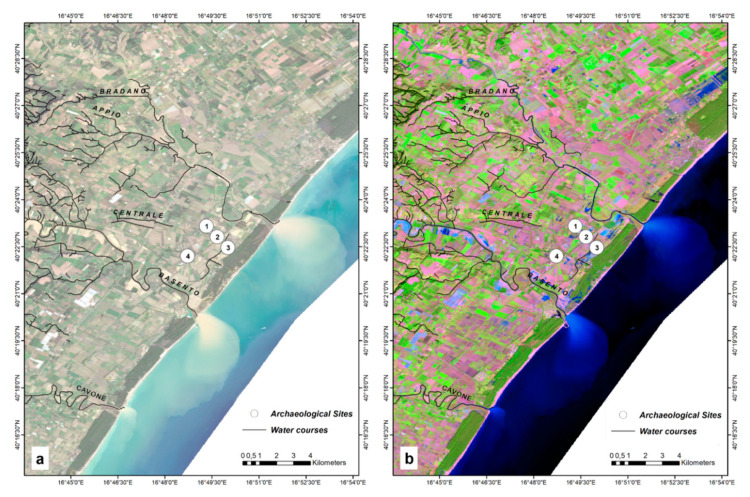
Localization of the region of interest. In the background, the (**a**) natural color (Red = Band 4; Green = Band 3; Blue = Band 2) and (**b**) vegetation analysis (Red = Band 6; Green = Band 5; Blue = Band 4) of Landsat 8 C2 L2 Imagery data acquired on 6 December 2013. Archaeological Sites: 1. Metaponto excavation; 2. Castrum; 3. Ancient remains; 4. Castello Torremare.

According to Lacava et al. [91], a major flood happened on 1–2 December 2013 which involved all of the rivers. Since the flood in 2013 happened before launching the Sentinel 1 and 2, this event was studied by the first cloud free Landsat 8 Operational Land Imager (OLI) and Thermal Infrared (TIRS) Collection 2 Level-2 Science Products 30-m multispectral data (on 6 December 2013). The RGB natural color (Red = Band 4; Green = Band 3; Blue = Band 2) (Figure 7a), and the RGB vegetation analysis (Red = Band 6 SWIR 1; Green = Band 5 NIR; Blue = Band 4 Red) (Figure 7b) of the acquired image were generated to give the overall situation of the study area.

**Figure 8:**
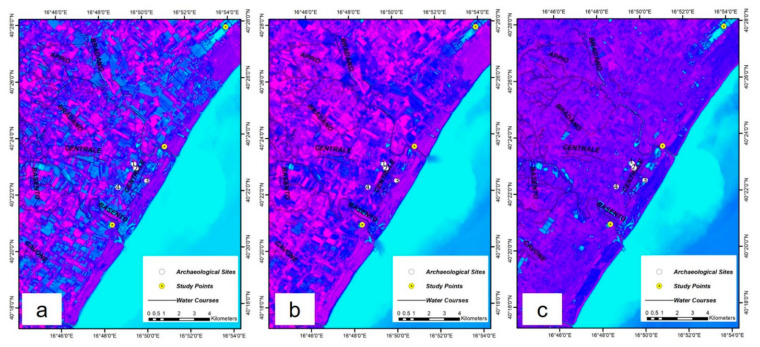
The raster maps of the (**a**) Landsat 8 Normalized Difference Vegetation Index (NDVI), (**b**) Normalized Difference Water Index (NDWI2), and (**c**) Modified Normalized Difference Water Index (MNDWI), for the study area relating to the 1–3 December 2013 flood. Archaeological Sites: 1. Metaponto excavation; 2. Castrum; 3. Ancient remains; 4. Castello Torremare.

Figure 8 represents the raster maps of the Landsat 8 NDVI, MNDWI, and NDWI2 indices for the study area relating to the December 2013 flood as generated by GEE code.

**Figure 9:**
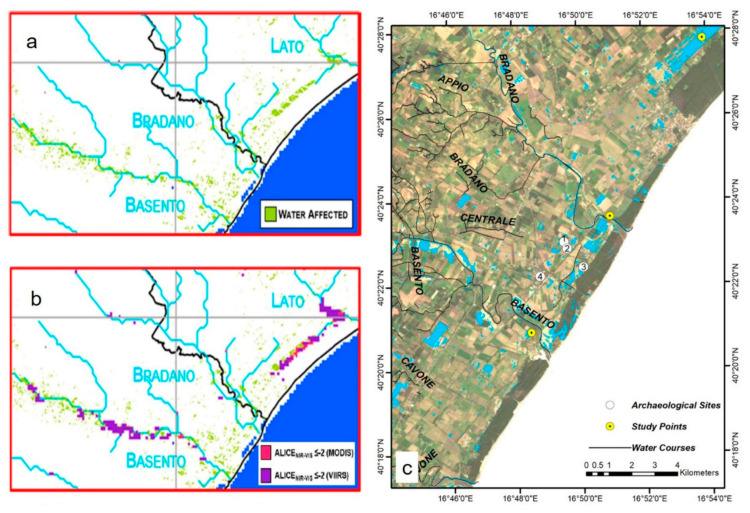
The flood map of the study area based on (**a**) thresholding the Landsat 7 NDVI raster on 5 December 2013; (**b**) anomalous pixels detected by RST-FLOOD algorithm on 5 December 2013 for Moderate-Resolution Imaging Spectroradiometer—MODIS (pink) and for Visible Infrared Imaging Radiometer Suite—VIIRS (violet); and (**c**) thresholding the Landsat 8 MNDWI raster on 6 December 2013. Figure 9a,b has been adopted from Lavaca et al. [68]. Archaeological Sites: 1. Metaponto excavation; 2. Castrum; 3. Ancient remains; 4. Castello Torremare.

According to Figure 8, MNDWI had the best performance to identify flooded areas. Thus, the flood map of the study area was generated by thresholding the raster histogram of Figure 8a into flooded and non-flooded categories (Figure 9). Additionally, the results of Lavaca et al. [68] from the same flood were added to Figure 9 to evaluate the results.

As can be seen in Figure 9, not only the flood map obtained from Landsat-8 MNDWI has a good consistency with those of Landsat-7 NDVI (Figure 9a) and MODIS and VIIRS RST-FLOOD algorithm (Figure 9b), but also it outperforms them considering the layout of the stream network and the spatial resolution of the maps (Figure 9c).

**Figure 10:**
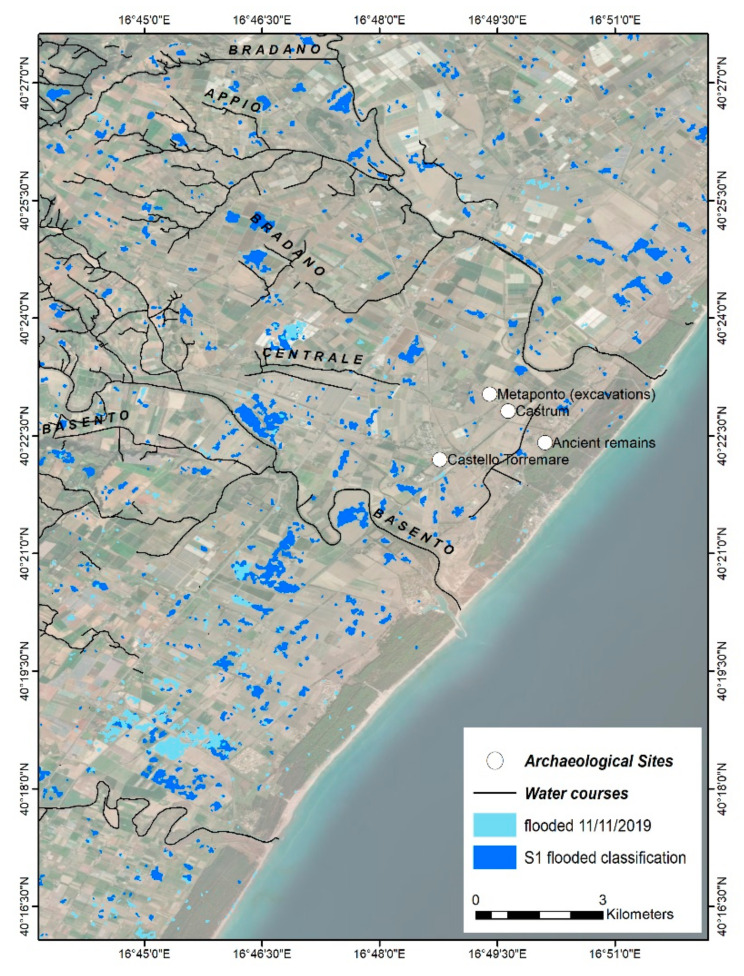
Sentinel-2 NDWI classification on 11 November 2019 (light blue) and Sentinel-1 before-and-after difference classification (dark blue). The image finds a useful comparison in Figure 8 of [65].

In another study, Bentivenga et al. [65] studied a recent flood in 11 November 2019 with the Sentinel 1 SAR images that was not due to the overflow of the main streams, but rather to the failure of the reclamation channels. In this case, the flood event was investigated by both Sentinel 1 and 2 images to provide a better vision of the event. The NDWI map of the study area was generated by Sentinel-2 image on 11 November and pixels with the negative values were classified as flooded areas. Moreover, the difference in the VH band between the first Sentinel-1 image before and after the flood was calculated and pixels with the difference greater than 1.15 were identified as flooded areas (Figure 10).

After delineating the most apparent flooded areas according to Figure 7, Figure 8 and Figure 9, the GEE was utilized to extract the variations of the Landsat-8 MNDWI values at five study points inside the flooded areas from 2013 to the present to investigate any apparent pattern in this parameter (Figure 9). Table 2 lists six major floods and their antecedent rainfalls in the Metaponto plain since 2013. Comparing Figure 8 and Figure 9 with the information in Table 2 reveals that the dramatic drop in the MNDWI matches with the occurrence of the three floods with the highest 30-day antecedent rainfall (i.e., October 2013, November 2013, and November 2018). Other floods could have been detected, but they might have been omitted due to the lack of cloud free images, or their antecedent rainfalls were not big enough to inundate study points.

**Figure 11:**
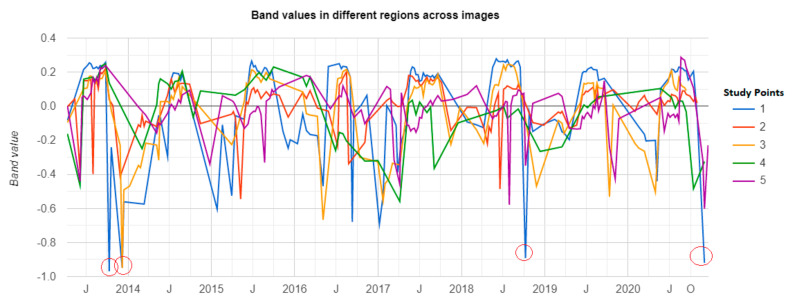
Time series of the MNDWI in five study points which are prone to flooding using Landsat 8 images in the Google Earth Engine (GEE).

**Figure 12:**
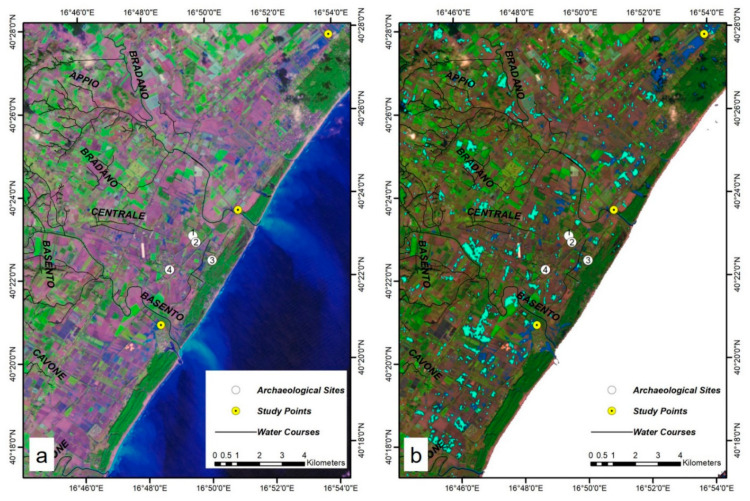
(**a**) Bands 6,5,4 RGB image of Landsat-8 image on 30 November 2020; and (**b**) flooded regions based on Landsat-8 MNDWI classification on 30 November 2020 (dark blue) and Sentinel-1 before-and-after difference classification (light blue). Archaeological Sites: 1. Metaponto excavation; 2. Castrum; 3. Ancient remains; 4. Castello Torremare.

Having the assumption of a minimum below −0.8 in the time-series of MNDWI as a condition for detecting a flood event, it is also visible in Figure 11 that there might be another major flood in November 2020. Thus, the Landsat-8 RGB image on 30 November 2020 was investigated for the possible flood event and as can be seen in Figure 12a, signs of flooding are evident in the study area. Figure 12b also reveals flooded areas as identified by thresholding the Landsat-8 MNDWI on 30 November 2020 as well as Sentinel-1 before-and-after difference classification. Sentinel-2 cloud coverage during the flood did not permit to extract flooded areas.

### 3.2. Forest Fire Mapping

**Figure 13:**
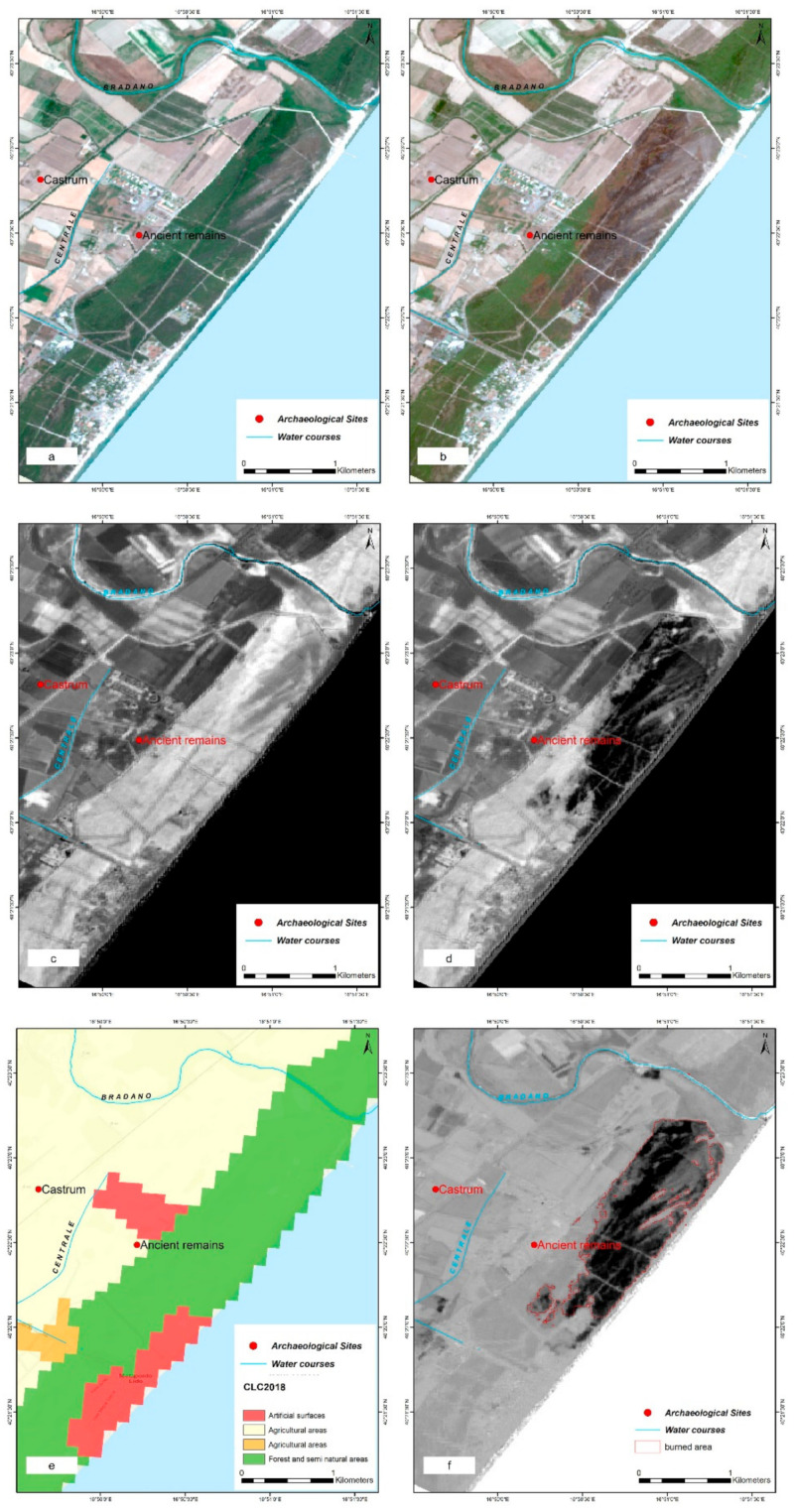
(**a**) S-2 RGB pre-fire image; (**b**) S-2 RGB post-fire image; (**c**) NBR greyscale pre-event; (**d**) NBR greyscale post-event; (**e**) Corine Land Cover 2018; (**f**) ∆NBR greyscale with perimeter of the burnt area automatically extracted by the system.

The use of GEE has allowed to automatize the data processing, herein discussed for the fire event occurred in 2017. After structuring the tool and inserting the coordinates of the fire, the images were created (Figure 13). The BA was identified by the darkest pixels within the ∆NBR (Figure 13d). The perimeter of the was generated as a shapefile (vector) and extracted automatically. The Corine land cover CLC 2018 was used to include the diverse land use and land cover types and allowed us (i) to remove false-positive related to changes occurred in agriculture area and to (ii) have information on the vegetation cover of the area affected by the fire event (Figure 13f).

**Figure 14:**
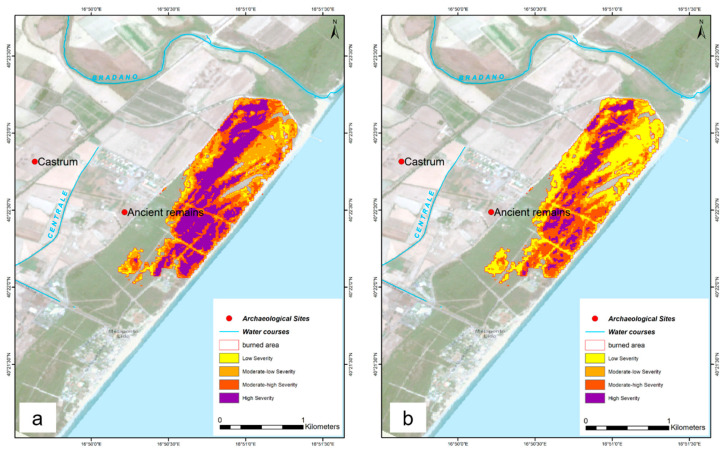
(**a**) S-2 threshold-based classification; (**b**) S-2 Unsupervised Classification.

The perimeter of the burned area was extracted by applying the canny edges algorithm in GEE. This process has allowed to apply a further mask, so that the severity levels are classified only within the burned area. The comparison made between the threshold classification and the unsupervised classification has produced the results shown in Table 3. The hectares affected are similar between the different classifications, but as can be seen in Figure 14, the categorization of burn severity level is different.

## 4. Discussions

The discussion of the results is herein divided into two parts, in accordance with the theme, that is the mapping of flood and fire affected areas and to categorize the levels of burn severity of the fire event.

### 4.1. GEE Based Mapping of Flooding

A straightforward rapid methodology for flood mapping and monitoring of the Metaponto plain was presented based on free satellite data available on the Google Earth Engine cloud platform. Three latest major flood events (in 2013, 2019, and 2020) were investigated with the latest available satellites providing high spatiotemporal resolution data of the study area (as Sentinel-1, Sentinel-2, integrated with Landsat-8 satellites). As the biggest flood event during the study period, December 2013 flood was selected to identify the maximum inundation area using Landsat-8 data, which was the only operating satellite at that time. Three thematic spectral indices that are capable of capturing water surfaces (i.e., NDVI, NDWI, and MNWDI) were calculated based on the recorded values at different Landsat-8 OLI bands. Comparing the raster maps of these indices with the Landsat-8 RGB image, and with the results of a previous study on the same flood using Landsat-7 ETM; revealed that NDVI is sensitive to water bodies while overestimating inundated areas in highly saturated bare soils, NDWI does not have this problem but is not sensitive to shallow inundated areas and MNWDI can reasonably delineate all inundated areas. Thus, the maximum inundation map of the Metaponto plain was created by classifying the MNDWI raster into flooded and non-flooded areas based on an optimum threshold (MNWDI <−0.6). In the November 2019 flood, only images from Sentinel-1 and 2 were available providing a good case for studying two different radar and multispectral techniques in performing flood mapping. The Sentinel-2 image had been taken at 09:43 of 11 November 2019 (the later images were not suitable) and it seemed that the flood was still at the early stages and the inundation had not been started yet. Therefore, MNDWI did not detect much inundation in the region, while NDWI detected some areas that seemed to be eventually inundated in the later stages of the flood. Sentinel-1, on the contrary, had good temporal coverage of the event and detected inundated areas using the before-and-after change detection method. This highlights the importance of radar data to enhance the flood mapping results. With the identification of flood-prone areas, it was possible to monitor spectral water indices to see if they can detect further floods with the near real-time domain. Inspecting the time series of MNDWI from Landsat-8 data in a number of inundated areas showed that determining global minima in the time series could be related to major flood events. To validate this assumption, such minimum in November 2020 was inspected and a flood event was successfully detected.

### 4.2. GEE Based Mapping of Burnt Areas and Severity

This paper evaluated the capability of a methodology based on Sentinel-2 images to map both burnt areas and burn severity close to the archaeological area of Metaponto. The methodology herein proposed was optimized through the use of the GEE cloud computing platform. The literature generally reports that, compared to other satellite data as Modis or TM, S2 offers higher accuracy in the discrimination and mapping of BA [112,123], that is relevant for the detection of small fire and for the monitoring of complex, heterogeneous, and fragmented ecosystems [125], typical of the Mediterranean area [135]. In addition, there are several techniques and methodologies for BA mapping. One of the most used procedures concerns bi-temporal images that combine algebraic operations [136], such as the calculation of the spectral index (NBR), and the pre- and post-fire temporal differences between them (ΔNBR) to improve the detection of the fire induced changes in vegetation cover.

GEE platform was essential at all steps of the processing, starting from the image visualization obtained in a few seconds, useful to select the most suitable pre- and post-fire images, without having to download or request them through the Copernicus Hub, provided by the European Space Agency (ESA), when the images are not online. Moreover, the results obtained showed that the process of BA identification and fire severity calculation, in different ways, can be done very quickly thanks to the computational power of the GEE platform. Similarly, in addition to the processes on satellite data of supervised or rule-based classification, machine learning and unsupervised classification processes can also be integrated, with remarkable results. BA mapping allows the visualization of fragile areas that are sensitive to fire damage, especially if these are conditioned by several elements such as the morphology of the territory, natural components of combustion (vegetation, soil moisture), and meteorological characteristics [137]. Above all, the results showed the great potential of GEE tool and its useful role in risk and damage management and monitoring. The methodology also showed promising results for BA mapping, showing its potential to support further assessment of the environmental impacts of BA at regional, national, and global levels, and to support fire risk management. This is particularly relevant for the preservation of high value heritage as natural and cultural properties and archaeological areas, that are unique and cannot be replaced if lost. Moreover, the impact of fire events can also affect the tourism sector and in turn the local economy as occurred in the Ionian coast of the Basilicata region (southern Italy) where the fire of 2017 also impacted the camping area which was evacuated for security reasons and also to facilitate the extinguishment of the fire.

## 5. Conclusions

The recent developments of both satellite sensors and data availability have attracted increasing attention for a wide spectra of applications including the monitoring of threats to cultural sites. In this paper, we focused on the use of open data and free technologies to set up an operational system to support risk management and in particular, the post event crisis management when timely, updates synoptic information are needed to better understand the spatial and temporal scale of the damage, and improve the mitigation strategy necessary immediately after the events. The most common traditional approaches addressed to the cultural heritage vulnerability are usually only focalized on the site itself, without considering the entire context at a landscape level, today considered as a critical priority of the preservation policies. In this context, remote sensing and Earth Observation technologies can provide open data and useful information systematical updated and available at diverse spatial and temporal scales. Several satellite platforms are currently in orbit and capture a wide variety of data, thus enabling multitemporal, multisensor, and multiscale analyses.

Today, the main critical, challenging aspect is the large amounts of EO BIG data—more than we can handle and with unimaginable increasing trend in the next future—and a lack of effective methods to automatically extract relevant and useful information. Big and open EO data (as, for example, those from Copernicus) offer big opportunities and big challenges, also linked, in the case the monitoring and management of threats to cultural sites also with the need of heritage managers to establish priorities and make choices to optimize the use of the available resources. Undoubtedly, EO has been widely recognized as useful tools for risk monitoring, even if there are still open challenges as the integration of diverse data source, the efficient storage, and data processing along with the assessment of the results obtained from EO, and also the possibility to develop a simplified approach which does not require elaborate expertise for implementation that can be set for heritage managers, as suggested by ICROMM [1].

The utilization of GEE enables us to overcome the drawbacks related to the data storage and computation facilities being that it is a free cloud platform very useful for a prompt implementation of different algorithms and analyses while removing the need for the prolonged steps of downloading and preprocessing the data. Moreover, as already tested in previous studies and herein proven, the methodology, once structured into a robust script, can be re-applied and scaled in different contexts and domains, making GEE great for cross-platform analysis. These advantages can be also coupled with the possibility to set user friendly interfaces making the GEE promptly ready also for operational applications. The outputs from the analysis herein conducted clearly pointed out that the set of tools offered by GEE is extremely useful for assessing and mapping fire and flood affected areas with the possibility to integrate several data sets in a rather simple way. This is possible without the need of having expensive hardware and software tools for the storage and computation which is definitely the greatest strength of this GEE. In addition, the GEE tool has proven to be extremely versatile in many ways, offering multi-sensor/platform data that can be processed through simple JavaScript programming suitable to several contexts and needs. In addition, any code produced in GEE can be easily disseminated through official Google channels, direct links, or repositories and, via API can be easily linked to GIS services, allowing anyone to reuse and adapt it on different scales and situations.

Using combined data, as herein proposed, is possible to overcome the limits related to a single type of sensor (optical), in favor of a combined use with other sensors such as synthetic aperture radar (SAR) [93,138], whose data are implemented in the GEE datasets. Moreover, the setup of a user-friendly interface makes GEE also very attractive for non EO experts as site manager and end user in charge of cultural sites for managing risks. In particular, the availability of weekly updated satellite images can timely support the mapping of damage from natural and manmade risks relevant and critical information for setting up damage mitigation strategies and support the preservation of areas and landscape rich in cultural and natural heritage. In particular, as highlighted in this paper, the possibility to timely map the extension of the areas affected by flood and fire and to categorize the levels of damage can suitably support the post event actions and better understand the spatial and temporal scale of the damage, thus improving the risk management and the preparedness plan and strategy.

## Figures and Tables

**Table 1:** The ∆NBR burn severity categories, according to the United States Geological Survey (USGS) categorization.

∆NBR	Burn Severity
≤−0.25	High post-fire regrowth
−0.25 to −0.1	Low post-fire regrowth
−0.1 to 0.1	Unburned
0.1 to 0.27	Low-severity burn
0.27 to 0.44	Moderate-low severity burn
0.44 to 0.66	Moderate-high severity burn
>0.66	High-severity burn

**Table 2:** Flood records in the Metaponto region along with their antecedent rainfalls that caused them. Adopted from [99].

Date	River Basin	Antecedent Rainfall	
		1 Day	30 Days
6–8 October 2013	Bradano, Basento	122	139.8
30 November–2 December 2013	Bradano, Basento, Cavone, Agri, Sinni	142	325
4–5 October 2014	Bradano, Basento, Cavone, Sinni	96.2	121.2
16–18 March 2016	Bradano, Basento, Cavone, Agri, Sinni	12.4	101.8
22–23 October 2018	Cavone, Agri, Sinni	20.8	239.2
11–12 November 2019	Bradano, Basento, Cavone, Agri, Sinni	37	51.8

**Table 3:** Comparison of fire severity levels between the classifications.

Classification Type	High Severity	Moderate–High Severity	Moderate–Low Severity	Low Severity
Threshold-based	64.8 ha	45.55 ha	44.63 ha	16.71 ha
Unsupervised	39.23 ha	47.8 ha	31.96 ha	52.7 ha
